# Less haste, less waste: on recycling and its limits in strand displacement systems

**DOI:** 10.1098/rsfs.2011.0106

**Published:** 2012-02-15

**Authors:** Anne Condon, Alan J. Hu, Ján Maňuch, Chris Thachuk

**Affiliations:** Department of Computer Science, University of British Columbia, Vancouver, British Columbia, Canada V6T 1Z4

**Keywords:** DNA computing, strand displacement systems, strand recycling

## Abstract

We study the potential for molecule recycling in chemical reaction systems and their DNA strand displacement realizations. Recycling happens when a product of one reaction is a reactant in a later reaction. Recycling has the benefits of reducing consumption, or waste, of molecules and of avoiding fuel depletion. We present a binary counter that recycles molecules efficiently while incurring just a moderate slowdown compared with alternative counters that do not recycle strands. This counter is an *n*-bit binary reflecting Gray code counter that advances through 2^*n*^ states. In the strand displacement realization of this counter, the waste—total number of nucleotides of the DNA strands consumed—is polynomial in *n*, the number of bits of the counter, while the waste of alternative counters grows exponentially in *n*. We also show that our *n*-bit counter fails to work correctly when many (**Θ**(*n*)) copies of the species that represent the bits of the counter are present initially. The proof applies more generally to show that in chemical reaction systems where all but one reactant of each reaction are catalysts, computations longer than a polynomial function of the size of the system are not possible when there are polynomially many copies of the system present.

## 1. Introduction

DNA strand displacement is a form of chemical reaction in which one or more single-stranded DNA molecules—the reactants—bind to a multi-stranded complex, thereby displacing other single-stranded molecules—the products. DNA strand displacements are important and versatile reactions that have already supported wet-lab simulation of logic circuits and DNA walkers and can in principle support general purpose computation in an energy-efficient manner [1–9]. Such DNA strand displacement reactions, and chemical reactions more generally, typically consume strands or reactants at all reaction steps. Catalyst strands are an exception in that they are not consumed during the course of a reaction, but are recycled to perform the same operation multiple times.

Can chemical reactions and their strand displacement system realizations recycle strands in more general ways? We show that the answer is yes: we describe chemical reaction system computations and their strand displacement realizations in which recycling of strands significantly reduces waste and avoids fuel depletion while incurring just a moderate slowdown relative to comparable computations that do not recycle strands. Thus our title: less haste, less waste. Our recycling computations are binary counters—simple and yet fundamental constructs in computation. A new feature of our strand displacement constructions is a mutex synchronization primitive, which ensures that reactions proceed atomically in the sense that all products of one reaction have been released before the next starts. The second contribution of the paper is to demonstrate a limit to recycling: recycling is not possible in certain classes of chemical reaction systems, which include certain classes of strand displacement systems that should work correctly even when many copies of the initial state of the system are present in the same environment.

The rest of this introduction illustrates the concept of strand recycling and gives an overview of our results and related work. Sections 2 and 3 then provide the technical details of the strand-recycling counters and the limits of recycling.

### 1.1. On the potential for strand recycling

**Figure 1.**
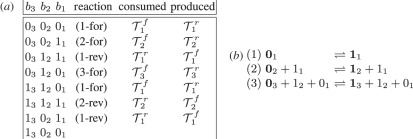
(*a*) Enumeration of counter states (left three columns), reaction that advances the state from one row to the next and its direction—forward (for) or reverse (rev)—and sets of transformer molecules consumed and produced. (*b*) The chemical reaction system for a 3-bit binary reflecting Gray code counter. Species that appear on just one side of the reaction are shown in boldface, which correspond to the bit that changes value as a result of the reaction. To ensure correctness, additional ‘catalyst’ species appear on both sides and the corresponding bits are unchanged. At any step, only one reaction is applicable to advance the counter, although since the reactions are reversible the counter could also retreat to its previous value.

We illustrate the concept of recycling using a 3-bit counter that is specified as a chemical reaction system—details of a strand displacement implementation are in §2. This binary reflecting Gray code counter [10] follows the sequence of bit values shown in the left column of figure 1*a*. It advances in such a way that exactly one bit changes at each step and gets its name from the following property: if the states of the counter are written in a column starting from 0_*n*_0_*n*−1_ … 0_1_ and a line is drawn just below row 2^*i*−1^, where bit *i* changes from 0_*i*_ to 1_*i*_, then in the next 2^*i*−1^ rows the values of the low order *i* − 1 bits are the reflection of those above the line. For example, consider bits *b*_2_ and *b*_1_ of the 3-bit sequence in figure 1*a*: the last four rows are a reflection of the first four rows. We call the resulting sequence of states the *Gray code sequence*.

Figure 1*b* gives the chemical reaction system for this counter, which we call GRAY. The state of the 3-bit GRAY counter is determined by three *signal* molecules, one per bit. The presence of a single copy of signal molecule 0_*i*_ denotes that the *i*th bit has value 0, while the presence of a single copy of 1_*i*_ denotes that the *i*th bit has value 1. The initial counter state is 0_3_0_2_0_1_ and the reactions ensure that exactly one of 0_*i*_ and 1_*i*_ is present at any time. The counter advances through application of the three reversible chemical reactions (1–3) of figure 1*b*. Each row of the table in figure 1*a* lists the reaction needed to produce the subsequent row; for example, the counter advances from 0_3_0_2_1_1_ to 0_3_1_2_1_1_ via reaction (2) in the forward direction (2-for).

**Table 1.** Comparison of *n*-bit counter implementations. The GRAY and GRAY-FO counters are described in §2. The Qian–Soloveichik–Winfree (QSW) counter is based on the simulation of stack machines by strand displacement reactions of Qian *et al.* [11].

properties	GRAY	GRAY-FO	QSW [11]
reaction order	**Θ**(*n*)	**Θ**(1)	**Θ**(1)
consumption (waste)	**Θ**(*n*^3^)	**Θ**(*n*^3^)	**Θ**(2^*n*^)
expected time (haste)	**Θ**(*n*^3^2^2*n*^)	**Θ**(*n*^3^2^2*n*^ )	**Θ**(2^2*n*^)

In realizing these reactions with strand displacement systems (see §2), additional *transformer* strands that are not shown in the chemical reaction system are consumed and produced. For example, transformers might serve to ensure that all reactants are available before any product is produced, or may be side-products of a reaction. Suppose that a set of strands *𝒯*_*i*_^*f*^ is consumed and a set *𝒯*_*i*_^*r*^ is produced when reaction (*i*) takes place in the forward direction; conversely *𝒯*_*i*_^*r*^ is consumed and *𝒯*_*i*_^*f*^ is produced when reaction (*i*) takes place in the reverse direction.

The key point is that in most of the rows, the signal molecules and transformer strands that are consumed were produced by reactions of earlier rows and are thus recycled. For example, in the third step the counter advances from state 0_3_1_2_1_1_ to 0_3_1_2_0_1_, uses reaction (1) in the reverse direction (1-rev) and consumes the signal molecule 0_1_ and the set of transformer strands *𝒯*_1_^*r*^ that were produced in the first step, thereby recycling *𝒯*_1_^*r*^. Only in the three rows 0_3_0_2_0_1_, 0_3_0_2_1_1_ and 0_3_1_2_0_1_—precisely those rows when a reaction occurs for the first time—the molecules consumed are not produced in earlier rows. In contrast, a chemical reaction system for a standard binary counter produces waste molecules at every step and these waste molecules are never recycled in subsequent steps.

Recycling in DNA strand displacement systems offers the potential of supporting energy-efficient DNA computations in which the waste, or number of strands consumed, is logarithmic in the length of the computation. Systems that recycle molecules do not use fuel, i.e. large concentrations of certain transformer species that bias reactions in one direction, and so are not prone to problems of fuel depletion or fuel leakage. However, such advantages come at a price: our counter proceeds somewhat more slowly—is less hasty—than comparable fuel-driven strand displacement counters. The slowdown is due in part to the fact that reactions are used in both directions. Thus, our GRAY counter is not biased to advance towards the final state but rather performs an unbiased random walk, both advancing and retreating, ultimately reaching the final state. We also describe a counter (GRAY-FO) that uses reactions of fixed order, i.e. the maximum number of reactants and products in any reaction are fixed, independent of the number of counter bits.

Table 1 summarizes properties of our counters and compares with another counter, which we call Qian–Soloveichik–Winfree (QSW), based on the work of Qian *et al.* [11] (see §1.3). The properties considered are (i) *order* or max number of reactants or products of chemical reactions that describe the counter, (ii) *waste* or total number of nucleotides needed to implement the counter, and (iii) *haste* or expected time for the counter to reach a designated final state from its initial state when the volume equals the waste. We describe how order, waste and haste grow as a function of *n*, the number of counter bits. Here and throughout the paper, we use *O*-notation and **Θ**-notation for this purpose. Formally, if *f*(*n*) and *g*(*n*) are non-negative functions, we say that *f*(*n*) is *O*(*g*(*n*)) if for all *n* above some size, *f*(*n*) is bounded above by a constant times *g*(*n*), i.e. *f*(*n*) ≤ *cg*(*n*) for some constant *c*. A function *f*(*n*) is **Θ**(*g*(*n*)) if *f*(*n*) is *O*(*g*(*n*)) and in addition, *f*(*n*) is bounded below by a (typically different) constant times *g*(*n*). That is, *f*(*n*) is **Θ**(*g*(*n*)) if *c*′*g*(*n*) ≤ *f*(*n*) ≤ *cg*(*n*) for some constants *c* and *c*′ and all sufficiently large *n*. Our use of **Θ**-notation enables us to distinguish between large differences in the growth of two functions without getting bogged down in details. For example, we will show that the waste of the GRAY counter is **Θ**(*n*^3^), meaning that the number of nucleotides of waste molecules needed to implement an *n*-bit counter grows roughly proportionally to *n*^3^, which is markedly less than the exponential (**Θ**(2^*n*^)) growth of waste molecules for the QSW counter.

Our *n*-bit binary reflecting Gray code counter, GRAY, uses reactions of maximum order **Θ**(*n*), generates only **Θ**(*n*^3^) waste and uses expected time **Θ**(*n*^3^2^2*n*^) to reach the final state. Our GRAY-FO counter improves on the GRAY counter in that the reaction order is **Θ**(1). The QSW counter also has reaction order **Θ**(1) and has expected time **Θ**(2^2*n*^), which is somewhat better than the expected time needed by our counters. However, the QSW counter generates **Θ**(2^*n*^) waste, exponentially worse than our counters. All three counters are deterministic in that they advance and retreat through a predetermined linear ordering of states.

### 1.2. On the limits of strand recycling

Our *n*-bit GRAY counter advances correctly through 2^*n*^ states because only single copies of initial species are present. In §3, we show that if **Θ**(*n*) copies of the initial species are present, then the counter does not advance properly in a very strong sense: the final state of the counter can be reached in just *O*(*n*^2^) chemical reactions, rather than using the intended sequence of 2^*n*^ reactions. We also prove more general limits on molecule recycling when multiple copies of the initial species are present, under some restrictions on the allowable chemical reaction systems. In particular, if the waste of such a chemical reaction system is logarithmic in the length of a valid computation, the system does not work correctly when polynomially many copies of the initial reactants are present.

### 1.3. Related work

Qian *et al.* [11] showed how to simulate a stack machine using strand displacement systems. A binary counter can be implemented via a stack machine; we call such a counter a QSW counter and we compare its properties and resources with our counters in table 1. We compare our results to a fuel-biased QSW counter as the unbiased version is slower and we also assume that all fuel must be initially present in the reaction volume—the counter operates in a closed system.

Cardelli [12,13] has shown how primitives that support concurrent models of computation, such as fork and join gates, can be implemented using strand displacement systems. Many of our techniques are similar to those of Cardelli's constructions: for example, our signal strands share a common toehold while the long domains are distinct, and we do not use branched structures. To effect an abstract chemical reaction with *i* reactants and *i* products, we use cascading of strand exchanges whereby the reactants are first absorbed (by transformer molecules) and products are then released by further strand exchanges. This order of events is similar to an *i*-way join followed by an *i*-way fork of Cardelli; it is similar also to the strand displacement realizations of *i*-way molecular reactions of Qian *et al.* [11]. A new feature of our constructions is the use of a *mutex* strand to ensure that the (*k* + 1)th reaction of a deterministic computation does not proceed until all products of the *k*th reaction have been produced.

Building on models of Winfree and Rothemund [14,15], Reif *et al.* [16] studied a tile-based graph assembly model in which tiles may both adhere to and be removed from a tile assembly. In their self-destructible graph assembly model, the removal of tiles allows for the possibility of tile reuse. The authors demonstrate that tile reuse is possible in an abstract tile model, via a PSPACE-hardness result. Doty *et al.* [17] showed a negative result on tile reuse for an irreversible variant of the model of Reif *et al.*

Kharam *et al.* [18] describe a DNA binary counter in which bit values are represented using relative concentrations of two molecule species. This is very different than our work in this paper, where the values of bits (0 and 1) are represented by the absence (or presence) of certain signal molecules.

## 2. GRAY: A binary reflecting Gray code counter

Here, we describe the chemical reaction system and strand displacement implementation of our GRAY counter, provide a proof of its correctness and analyse its expected time (haste) and space usage (waste). We show how it can be modified to use only bi-molecular reactions, resulting in our fixed-order GRAY counter: GRAY-FO.

### 2.1. Chemical reaction system for the GRAY counter

We generalize the 3-bit GRAY counter in §1.1 to *n*-bits. The counter state is represented by *n* *signal* molecules, one per bit. Presence of signal molecule *b*_*i*_ denotes that the *i*th bit has value *b*_*i*_, for *b* = 0 or *b* = 1. Initially, the state is 0_*n*_ … 0_2_ 0_1_ . Each possible state of the counter represents a value in the Gray code sequence. The counter is described abstractly by the following chemical reactions:

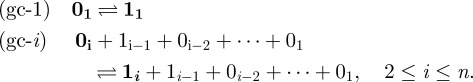



**Lemma 2.1.** *This chemical reaction system ensures that the GRAY counter, when in state v, can only advance to state v_*next*_, or retreat to state v_*prev*_, corresponding to the next or previous value in the Gray code sequence, respectively, if each reaction is atomic,*^1^ *and all initial signal molecules exist as single copies*.

*Proof.* First, observe that at any state of the system, for each *i*, exactly one of the signal molecules 0_*i*_ and 1_*i*_ is present (in an unbound state). Hence, at any state of the system, only two reactions can be applied: (gc-1) and (gc-*i*), where *i* is the smallest index such that signal molecule 1_*i*−1_ is present. Indeed, the reactions (gc-*j*), where 2 ≤ *j* < *i*, cannot be applied as, by the definition of *i*, signal molecule 0_*j*−1_ is present, and hence, 1_*j*−1_ is not present. Similarly, the reactions (gc-*j*), where *j* > *i*, cannot be applied as signal molecule 1_*i* −1_ is present, and hence, 0_*i*−1_ is not present. It follows that at any state of the system, the system can only progress in the forward or the backward directions.▪

### 2.2. Strand displacement implementation of the GRAY counter

**Figure 2.**
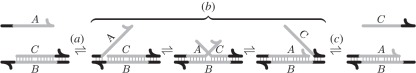
Strand displacement. (*a*) Toehold (black subsequence) of molecule *A* binds with its unpaired complement on molecule *B*. (*b*) Domain (grey subsequence) *δ* of *A* competes via a random walk process with the *δ* domain of *C* to bind with the complementary domain δ* of *B* until all bases of *A* are bound to *B*. (*c*) Toehold of *C* detaches from *B*, at which point it has been displaced.

Soloveichik *et al.* [7] showed that arbitrary chemical reaction systems could be realized by using DNA molecules as the chemical species and DNA strand displacement reactions to implement reactions among those species. We illustrate a simple, reversible version of strand displacement in figure 2. First, a subsequence of a single-stranded molecule *A* binds to a duplex *B*-*C* by forming base pairs with a short complementary subsequence of unpaired bases of *B*. The short subsequences are called *toeholds* and are the black segments of the molecules in figure 2. Then, in a random walk process (often referred to as branch migration), the remaining bases of *A* compete with *C* to form base pairs with *B* because both *A* and *C* contain an identical subsequence *δ* that is complementary to a subsequence of *B*. We refer to sequence *δ* as a *domain*. Once the *δ* domain of *A* has bound to its complement δ* of *B*, *C* remains bound to *B* by just a short toehold. The toehold bonds can break, thereby releasing *C*. (Of course, *A* may detach from the duplex *B*-*C* before *C* is released, in which case the reaction does not happen.) The reaction is reversible because *C* can bind to duplex *A*-*B* to displace *A* via the same principles. We refer to *A* and *C*, i.e. the molecules that are bound and released, as *signal molecules* and we refer to the duplexes *B*-*C* and *A*-*B* as transformer molecules. Abstractly, the overall process can be viewed as the reaction ***A*** ⇌ ***C*** where, abusing notation somewhat, we use *A* and *C* to denote abstract molecular species, rather than individual DNA signal molecules that realize these species and we ignore the transformers. Consistent with Soloveichik *et al.* [7], we assume throughout that if an environment contains DNA strands with different domains, say *δ* and *δ*′, then domains can be designed to be sufficiently different that a strand with domain *δ* never displaces a strand with domain *δ*′.

A strand displacement implementation of the GRAY counter requires a means to simulate its chemical reaction equations, which involve multiple reactants and products. Furthermore, the correctness of the counter is predicated on the assumption that each chemical reaction is *atomic*.

Qian *et al.* [11] proposed a construction—hereafter called the QSW construction—that is capable of simulating bi-molecular, and higher order, chemical reactions. Specifically, the construction can exchange a set of signal molecules (the reactants) for another set of signal molecules (the products) through a sequence of strand displacement events. Unfortunately, the construction does not simulate the higher order reaction atomically, because some product signal molecules can start initiating other reactions before all product signal molecules are produced. However, the strand displacements do occur in a fixed order and all reactant signal molecules are consumed before any product signal molecule is produced. We exploit this fact to simulate atomicity.

In particular, we borrow the concept of *transactions* from database and concurrency theory—a group of operations that either completes or does not complete in its entirety, and does not interfere with any other transaction. We implement transactions using a simple synchronization primitive: a *mutex*. A transaction must acquire the mutex in order to start, and releases it only when it completes. The state of our counter is defined only when the mutex is available. More precisely, let *μ* denote a single copy of a signal species representing the mutex. In any sequence of strand displacements representing a chemical reaction, *μ* is the first reactant to be consumed and the last product to be produced. Therefore, only one reaction (transaction) can be in progress at any given time. When *μ* is next available, either all strand displacements in the sequence took place and the counter is in a new state—the transaction succeeded—or the counter is in the same state and the configuration of all molecules is exactly the same prior to the reaction beginning—the transaction failed. Because each reaction is implemented as a transaction, it appears atomic and cannot interfere with other reactions.

**Figure 3.**
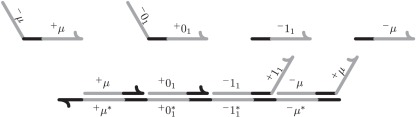
An example of signal molecules (top two left strands) and the transformer, consisting of auxiliary strands (top two right strands) and a saturated template strand (bottom complex) associated with the forward direction of **0_1_**⇌**1_1_**. In this and later figures, the Watson–Crick complement of a domain *x* is denoted by x* . The state of the system shown is 0_1_ .

We will use only one type of toehold, and therefore we will not label toeholds in the figures below. All signal molecules in the QSW construction are of the same form: a negative recognition domain ^−^*d*, followed by a toehold *t* and by a positive recognition domain ^+^*d*. The construction also uses auxiliary strands consisting of a single domain and a single toehold, and one template strand initially bound to signal molecules and auxiliary strands in such a way that no domain of the template is uncovered. We call a template strand with all domains bound to other strands, *saturated*. We refer to the saturated template complex and associated auxiliary strands, collectively, as a transformer. An example of the signal molecules and the transformer associated with the forward direction of the reaction **0_1_**⇌**1_1_** is given in figure 3.

**Figure 4.**
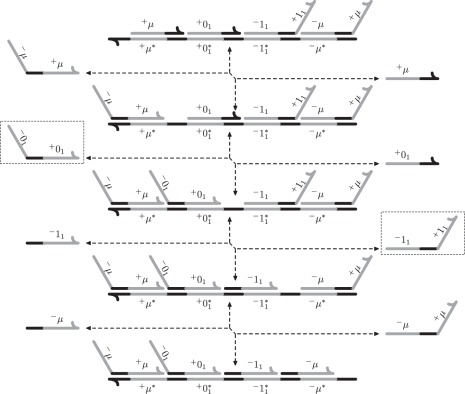
The sequence of strand displacement events for the reaction **0**_**1**_⇌**1**_**1**_.

As previously discussed, the reaction can only initiate if the signal molecule *μ* is present, and can only complete if all other reactants—in this case 0_1_, assuming a forward reaction—are available. An example of the sequence of strand displacements for the reaction **0_1_**⇌**1_1_** is given in figure 4. The reaction proceeds from top to bottom in the forward direction and from bottom to top in the backward direction.

**Figure 5.**
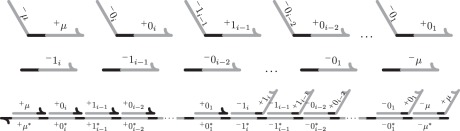
An example of the signal molecules and the transformer molecules for the *i*th reaction. The counter is in state *b*_*n*_ … *b*_*i*+1_ 0_*i*_ 1_*i*−1_ 0_*i*−2_ … 0_1_ .

The transformers that implement the *i*th reaction (gc-*i*) are a straightforward generalization of the first reaction. As before, the signal molecule *μ* must initiate the first strand displacement, and is not produced until the last strand displacement. The number of required intermediate strand displacement reactions is dependent on the number of reactants and products. Specifically, the *i*th reaction requires 2*i* + 2 strand displacements to complete. An example of the transformer for the *i*th reaction is given in figure 5.

### 2.3. Correctness

In the reactions of our counter, strand displacement should only happen when the toehold of the invading strand first binds to a free toehold, following which a domain of the invading strand displaces the bound domain of the strand being released. The invading and released domains should be identical. We say that such a strand displacement is *legal*. Illegal strand displacements can arise when the invading domain is different from the released domain; we call such displacements *mismatched domain displacements*. Illegal strand displacements can also arise due to blunt-end displacement, i.e. displacements where invading and released domains are identical but domain displacement is not preceded by the binding of a free toehold, or when more than one invading domain strand displaces the strand being released. Designing strands to ensure only legal displacements occur with high probability is beyond the scope of this paper. We will assume that only legal displacements occur.

**Lemma 2.2.** *The earlier mentioned strand displacement implementation of the GRAY counter ensures that all chemical reactions occur as transactions* (*and therefore appear atomic*), *assuming all strand displacements are legal*.

*Proof.* We argue by induction on the sequence of chemical reactions. Prior to any chemical reaction beginning, we require the following invariant to hold: (i) all template strands of all transformers are saturated, and require the mutex signal molecule *μ* to initiate the first strand displacement and (ii) there is exactly one available copy of *μ*. The invariant is trivially satisfied for the base case, when no reaction has yet occurred. Suppose the first *i* − 1 reactions appear atomic, and the invariant is satisfied. Without loss of generality, suppose the next attempted reaction involves the *k*th transformer.

Because we assume that all strand displacements are legal, no auxiliary strand or signal molecule representing the value of a digit can displace any strand in any transformer. Since there is exactly one available copy of the mutex signal species *μ*, that strand alone can initiate a reaction. Suppose the reaction is in the forward direction, as the reverse direction is symmetric. The signal molecule *μ* must initiate the first strand displacement by binding to the left end of the *k*th transformer's template strand. This begins the transaction. Note that there is another copy of *μ* sequestered at the right end of the template. When the signal molecule *μ* is once again produced, there are two cases to consider.

*Case 1.* If the copy on the right end of the transformer is released, then the transaction succeeded and the counter is in a new state. Furthermore, the invariant is preserved as (i) the *k*th transformer is saturated, and only a signal strand *μ* can initiate a new reaction on the right end of the template and (ii) exactly one signal molecule *μ* was produced as the final strand displacement.

*Case 2.* Otherwise, the original copy of *μ* was released, the transaction failed, and the counter is in the same state, satisfying the invariant, as any intermediate strand displacements must have been reversed prior to the original *μ* signal molecule being released.

Importantly, whether or not a transaction succeeds, while one is in progress no other reaction can be initiated because no other copy of signal species *μ* is available. Thus, all reactions are implemented as transactions and appear atomic.▪

### 2.4. Waste and haste analysis of the GRAY counter

Here, we analyse the waste—the total number of nucleotide bases of all species consumed and haste—expected time—of the GRAY counter as it advances from initial to final states. We assume single copies of the initial signal, transformer and mutex species. To analyse waste, we first count the number of bases required for all initial signal, transformer and mutex molecules.

**Lemma 2.3.** *The total number of nucleotide bases needed for a single copy of each initial signal, transformer and mutex species of the n-bit GRAY counter is* **Θ**(*n*^3^).

*Proof.* Each signal molecule 0_*i*_ and the initial mutex molecule *μ* is composed of a toehold and two long domains. The same is true of the molecules for states 1_*i*_ and the sequestered signal molecules *μ* that are part of the initial transformer species. There is an auxiliary transformer strand molecules consisting of one toehold and one long domain for each type of signal species. Similar to previous strand displacement implementations [7], we assume the **Θ**(*n*) domains of the signal species can be designed to have length **Θ**(*n*) to ensure only legal displacements occur for the duration of the counter. We choose the toehold length to be **Θ**(1) Because the domain length dominates the toehold length, the total number of bases in all signal species and auxiliary strands is **Θ**(*n*^2^).

The template strands in the sets *𝒯*_*i*_^*f*^ and 𝒯_*i*_^*r*^ have **Θ**(*i*) domains, which dominate their length, and thus the template strands have length **Θ**(*in*). Thus, the total number of bases in all transformer molecules in the system is 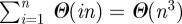.

Next, consider the expected time (haste) for the counter to progress from its initial to final states.

**Lemma 2.4.** *Assuming a single copy of each initial signal, transformer and mutex species, and that all strand displacements are legal and all reactions occur as transactions* (*appear atomic*), *the GRAY counter advances through the *2**^*n*^ *states of the Gray code sequence in* **Θ**(*n*^3^2^2*n*^) *expected time* (*haste*).

*Proof.* We assume that reactions occur in a volume of size **Θ**(*n*^3^), because this is the total number of bases of species in the system. Each strand displacement step involves interaction between two species and thus the rate of each strand displacement step is 1/**Θ**(*n*^3^).

First, consider the *shortest path* from the initial state to the final state. On this path, each order-*i* reaction is applied 2^*n*−*i*^ times and involves **Θ**(*i*) strand displacements. Thus, the total number of strand displacement steps along the shortest path is 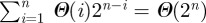.

Because each reaction is reversible, the system does not strictly follow the shortest path but rather proceeds as an unbiased random walk along this path. The expected number of steps for a random walk to reach one end of a length-**Θ**(2^*n*^) path from the other is **Θ**((2^*n*^)^2^) = **Θ**(2^2*n*^) [19]. Therefore, the expected number of strand displacement steps is **Θ**(2^2*n*^). Since each strand displacement step occurs at a rate of 1/**Θ**(*n*^3^), the overall expected time—the haste—is **Θ**(*n*^3^2^2*n*^). Note that the haste is polynomial in the **Ω**(2^*n*^) steps required to proceed through all 2^*n*^ unique states of an *n*-bit binary Gray code counter.▪

Combining lemmas 2.1–2.4, we have our first main result.

**Theorem 2.5.** *An n-bit binary reflecting Gray code counter can be implemented as a DNA strand displacement system that proceeds through the *2*^n^ unique states of the Gray code sequence in* **Θ**(*n*^3^2^2*n*^) *expected time* (*haste*) *and uses only* **Θ**(*n*^3^) *nucleotides* (*waste*).

### 2.5. A fixed-order implementation of the GRAY counter

An *n*-digit GRAY counter can perform a computation having length exponential in *n*, while only generating waste polynomial in *n*. However, it relied on template strands containing *O*(*n*) domains, each of length *O*(*n*), resulting in an overall length of *O*(*n*^2^) bases. Synthesis of long nucleic acid strands is challenging, and the fidelity of synthesized strands generally decreases as sequence length increases. For this reason, it is desirable to bound the length of all strands in the system to *O*(*n*) bases. We now briefly describe how a template strand from the GRAY counter consisting of 2*i* + 2 domains can be split into *i* + 1 template strands requiring four domains each, for any *i* > 1. The overall waste will only be increased by a constant, resulting in the same volume, and thus the same haste.

To simplify the description, we introduce some notation. Consider the (gc-i) reaction of the GRAY counter that has *i* reactants and *i* products:

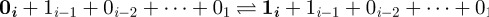



The previous implementation demonstrated that by using the QSW construction and introducing a mutex species *μ*—thus creating an order *i* + 1 reaction—chemical reactions occur as transactions and therefore appear atomic. Specifically, *μ* is first consumed, then 0_*i*_, then 1_*i*−1_ and so on. Likewise, after all reactants are consumed, 1_*i*_ is first produced, then 1_*i*−1_ and so on, until finally *μ* is produced. We denote a strand displacement implementation supporting a transaction of this type, which is initiated by consuming a mutex *α*, and terminated when producing a mutex *β*, by

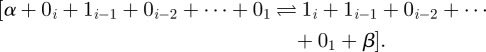



In the case of the GRAY counter, *α* = *β*= *μ* . Our goal is to convert this order *i* + 1 reaction into a cascade of *i* + 1 bi-molecular reactions, while preserving the appearance of atomicity. Using the earlier mentioned notation, we implement the following reaction cascade:

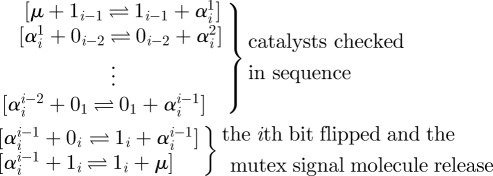



The overall transaction has been split into a cascade of sub-transactions. Each sub-transaction is implemented as a bi-molecular reaction using the QSW construction (figure 4). The first *i* − 1 sub-transactions check, in sequence, that all *i* − 1 catalysts are present. The mutex signal molecule *μ* is consumed during the first check. The last two transactions will first perform the bit flip and then releases the mutex signal molecule. Every sub-transaction, except the (*i* − 1)st and the *i*th, produces a unique mutex molecule that is required to initiate the next sub-transaction in the cascade. Upon successful completion of the first *i* sub-transactions in the cascade, the final sub-transaction occurs, producing the original mutex molecule *μ*, and thus finalizing the overall transaction. The implementation works in the reverse direction in a similar way with the exception that the bit is flipped first and the mutex signal molecule *μ* is released only after the presence of all catalysts have been verified. Using the earlier mentioned transformation for all higher order reactions in the original GRAY counter implementation results in a new, fixed-order counter, GRAY-FO.

## 3. Limits on molecule recycling in general chemical reaction systems

In this section, we show that certain classes of chemical reaction systems that efficiently recycle strands, or that can perform useful computations for time that significantly exceeds the number of signal molecules, cannot work properly when multiple copies of the initial signal molecules are present. In particular, our GRAY counters do not work in a multi-copy setting.

The underlying problem is the representation of the state of the system as specific *combinations* of signal molecules. If there are multiple copies of the system in the same reaction vessel—as would typically occur in a laboratory setting—then the states of the different copies may interfere with one another. To illustrate this point, we again consider the 3-bit GRAY counter. Initially, in a single copy of the construction, the signal molecules {0_3_, 0_2_, 0_1_} denote the state 0_3_0_2_0_1_. Consider a two-copy system where the initial set of present signal molecules is duplicated, yielding the multiset {0_3_, 0_3_, 0_2_, 0_2_, 0_1_, 0_1_}. As in the single copy case, assume reaction (1) occurs in the forward direction, followed by reaction (2) in the forward direction. The resulting multiset of signal molecules is {0_3_, 0_3_, 0_2_, 1_2_, 0_1_, 1_1_}. In the single copy case, we intend that reaction (1) in the reverse direction will occur next; however, given the current set of present signal molecule in the two-copy case, reaction (3) in the forward direction could instead occur, resulting in the multiset {0_3_, 1_3_, 0_2_, 1_2_, 0_1_, 1_1_}. At this point, a copy of every signal molecules is present, and any reaction can occur, in either direction. Furthermore, the single copy case required *at least* seven reactions to produce the final state 1_3_0_2_0_1_ , whereas the two-copy case can reach it in three. Crosstalk between the copies has broken the counter.

In the remainder of this section, we treat this problem formally. We define a *chemical reaction system* to be a tuple **C** = {*S*, *ℛ*, *S*_0_, *s*_end_}, where

— *𝒮* is a set of signal species.
— *ℛ* is a set of *reaction equations*, where each *R* ∈ *ℛ* is an ordered pair of multisets of signal molecules. Intuitively, a reaction equation *R* = (*I*, *P*) consumes the signal molecules in *I* as *inputs* and produces the signal molecules in *P* as *products*. Our formalism is directional to allow modelling non-reversible reactions; a reversible chemical reaction is modelled as two separate elements of *ℛ*, i.e. (*I*, *P*) and (*P*, *I*).
— *S*_0_ is a multiset of signal molecules initially present.
— *s*_end_ ∈ *S* is a signal molecule denoting the end of computation.^2^

**Example 3.1.** Let us describe the 3-bit GRAY counter as a chemical reaction system. The set of signal species is *S* = {0_1_, 0_2_, 0_3_, 1_1_, 1_2_, 1_3_, *s*_end_}, where *s*_end_ denotes the end of computation. The initial multiset is *S*_0_ = {0_1_, 0_2_, 0_3_}. Finally, we have the following set of reaction equations:

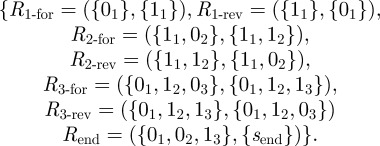



These reactions, with the exception of the last one, formally define the reactions shown in figure 1*b*.

An *x-copy* version of **C**, denoted **C**^(*x*)^, is obtained by duplicating the initial multiset *S*_0_ *x* times, i.e. **C**^(*x*)^ = 〈*S*, *ℛ*, *S*_0_^(*x*)^, *s*_end_〉, where *S*_0_^(*x*)^ is a multiset consisting of *x* copies of *S*_0_.

We formalize computations in **C** in the natural manner: let *ρ* be a sequence of reactions *R*_1_, *R*_2_, …, *R*_*m*_ from *ℛ*, where each *R*_*i*_ = (*I*_*i*_, *P*_*i*_). We define *ρ* to be a *trace* of **C** if *ρ* induces a corresponding sequence of multisets *S*_0_, *S*_1_, …, *S*_*m*_, with *S*_0_ being the multiset of initial signal molecules in **C**, and for all 1 ≤ *i* ≤ *m*, we have both *I*_*i*_ ⊆ *S*_*i*−1_ and *S*_*i*_ = *S*_*i*−1_ − *I*_*i*_ + *P*_*i*_. (We use ‘−’ and ‘+’ to denote multiset subtraction and union.)

**Example 3.1 (continued).** The shortest trace producing *s*_end_ is the sequence of reactions

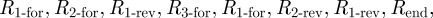

which induces the following sequence of multisets:

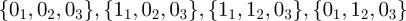

and

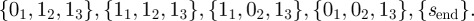



We use *B*_*s*_ to denote the *bandwidth* of signal species *s*, i.e. the maximum number of copies of *s* that appears in a multiset *I* of any reaction (*I*, *P*) ∈ *ℛ*, and we use *B*_**C**_ to denote the *bandwidth* of **C**, i.e. the sum of bandwidths of all signal species in *S*.

The next definitions help delineate the class of chemical reaction systems for the main result of this section. For a reaction equation *R* = (*I*, *P*), consider the signal molecules in *I* − *P*. We dub these input signal molecules *proper*. The other signal molecules, in *I* ∩ *P*, function as catalysts—they are necessary for the reaction, but not consumed. We define a set of reactions to be *k-proper* if *k* is the maximum number of proper inputs of all reactions in the set. (Since *I* is a multiset, each proper copy of the same input molecule in *I* contributes one to the overall count of proper inputs.) Note that the GRAY counter system is 1-proper. Finally, we use |*X*| to denote the number of elements in *X* with multiplicities for any multiset *X*.

**Theorem 3.2.** *Let* **C** = 〈*S*, *ℛ*, *S*_0_, *s*_end_〉 *be a* 1-*proper chemical reaction system. If there exists a trace that produces* *s*_end_ *in* **C**, *then for the x-copy chemical reaction system* **C**^(*x*)^ *with x ≥ B*_**C**_ + 1, *there exists a computation that produces* *s*_*end*_ *in at most* (*B*_**C**_ + 1)*B*_**C**_/2 + 1 *steps*.

*Proof.* Let *ρ* = *R*_1_, …, *R*_*m*_ be a trace for a computation that produces *s*_end_ in the last step in the (single-copy) system **C** and let *S*_0_, …, *S*_*m*_ be the corresponding sequence of multisets of signal molecules. Let *S*′ be the multiset of signal molecules obtained by including *w*_*s*_ copies of each *s* ∈ *S*, where *w*_*s*_ ≥ 0 is the maximum number of copies of signal molecule *s* that appears in a multiset *I* of any reaction (*I*, *P*) of the sequence *ρ*. Note that |*S*′| = *B*_**C**_. Let *k* = |*S*′ − *S*_0_|. Note that *k* ≤ *B*_**C**_. The goal is to produce all signal molecules in the multiset *S*′ − *S*_0_, so that we can apply the last reaction of *ρ* and produce *s*_end_.

We construct a trace of the appropriate length for the multi-copy system from the trace *ρ* for the single-copy system. The high-level structure of the proof is as follows. First, we project out from *ρ* the *k* reactions, in order, that first produce each of the molecules in the multiset *S*′−*S*_0_. From that sequence, we build a trace of the multi-copy system that is the concatenation of *k* phases. Each phase adds one more signal molecule to the multiset of signal molecules present, preserves the presence of all signal molecules previously produced and ‘consumes’ one copy of the initial signal molecules in *S*_0_. We will show that the *j*th phase is at most *j* reactions long; so the total length of the trace producing *S*′ − *S*_0_ is bounded by 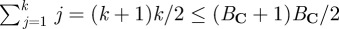.

We now formalize the construction of the *k* phases. Define the first appearance of the *c*th copy of signal molecule *s* to be in *S*_*i*_ if there are at least *c* copies of *s* in multiset *S*_*i*_ and less than *c* copies of *s* in each of *S*_0_, *S*_1_, …, *S*_*i*−1_. Let *s*_1_, …, *s*_*k*_ be the sequence of signal molecules (with multiplicities) from *S*′ − *S*_0_ in order of their first appearances in *S*_1_, …, *S*_*m*_ and let *R*_index_(*s*_*j*_) be the reaction in *ρ* that first produced this copy of *s*_*j*_. In other words, *R*_index_(*s*_*j*_) is the reaction that produced the first appearance of *s*_*j*_ (where *s*_*j*_ is the *c*th copy of some signal molecule *s*, for some *c*). The *k* phases will produce the signal molecules in *S*′ − *S*_0_ exactly in this order: signal molecule *s*_*j*_ will be produced in phase *j*. Each phase *j* will consist of several reactions: 0 to *j* which will produce *s*_*j*_ without removing any other signal molecule from set *S*′ − *S*_0_, but they can remove one signal molecule from *S*_0_, which is replenished by adding one new copy of *S*_0_ at the beginning of this phase. To find the sequence of these reactions, we will work backwards. Assuming *s*_*j*_ is not already present in the current multiset of signal molecules, we use reaction *R*_index(*s*_*j*__)__ to produce *s*_*j*_. As a result, we might have removed one of the signal molecules *s*_1_, …, *s*_*j*−1_, say *s*_*i*_. If that is the case, we repeat the process of producing signal molecule *s*_*i*_, i.e. repeating reactions of phase *i*.

The *k* phases are constructed to maintain three invariants.

— After the *j*th phase, the multiset of signal molecules contains the multiset {*s*_1_, …, *s*_*j*_}.
— The trace constructed so far has not relied on the existence of more than *j* copies of the initial signal molecules *S*_0_.
— For each *i* ≤ *j*, the *i*th phase has used at most *i* reactions.

The invariants are vacuously true initially (before any phases). Assuming they are true after *j* − 1 phases, we construct the *j*th phase as follows. If *s*_*j*_ is already present in the current multiset of molecules, we do nothing. Otherwise, the first reaction in the phase is *R*_index_(*s*_*j*_), the reaction that produced *s*_*j*_ for the first time. We know this reaction can be applied because all of {*s*_1_, …, *s*_*j*−1_} are available, as well as the *j*th copy of *S*_0_. This guarantees that the multiset now contains *s*_*j*_, and we have relied on only *j* copies of *S*_0_. However, because the system is 1-proper, the reaction consumed at most 1 input signal molecule. If the reaction consumed 0 molecules, or if the 1 molecule is in *S*_0_, the invariant is maintained and the phase ends. Otherwise, the reaction consumed some *s*_*i*_, where *i* < *j*. To restore *s*_*i*_ to the multiset, we repeat the sequence of reactions of the *i*th phase. Note that this is valid because the new copy of *S*_0_ was not yet used and all signal molecules required for phase *i* are still present. The number of reactions of phase *j* can be bounded by the number of reactions of phase *i* plus one. By the induction invariant, the *i*th phase requires at most *i* reactions and since *i* < *j*, the *j*th phase requires at most *j* reactions.

Concatenating the *k* phases produces a trace for the *k*-copy chemical reaction system **C**^(*k*)^, which produces all of {*s*_1_, …, *s*_*k*_} within (*k* + 1)*k*/2 reactions. If *s*_end_ is not in *S*′ − *S*_0_ = {*s*_1_, … , *s*_k_}, then *S*′ contains all inputs needed for the last reaction in *ρ* that produces *s*_end_. Thus, to produce *s*_end_, we might need one additional copy of the initial set and one additional step. Since *k* ≤ *B*_**C**_, the result follows.▪

The following two examples show that theorem 3.2 cannot be improved without altering the definition of chemical reactions systems. The first example shows that the bound on the number of steps is tight.

**Example 3.3.** Consider the following 1-proper chemical reaction system **E**_1_:

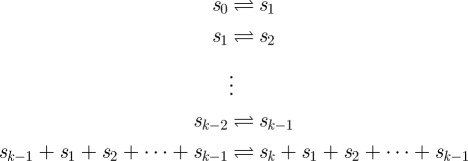

with the initial set containing *k* copies of *s*_0_. Consider the *x*-copy chemical reaction system **E**_1_^(*x*)^, where *x* is arbitrarily large. To produce *s*_*k*_, we need one copy of signal molecules *s*_1_, …, *s*_*k*−2_ and two copies of signal molecule *s*_*k*−1_. The number of steps needed to produce signal molecule *s*_*i*_ from *s*_0_, *i* < *k*, is *i*. Hence, the number of steps needed to produce all required signal molecules is 1 + 2 + …+ *k* − 1 + *k* − 1 = (*k* + 1)*k*/2 − 1. Thus, the number of steps needed to produce *s*_*k*_ is (*k* + 1)*k*/2, which meets the bound of theorem 3.2 because *k*=|*S*′ − *S*_0_|.

The second example shows that the assumption that the system is 1-proper is crucial.

**Example 3.4.** Consider the following 2-proper chemical reaction system **E**_2_:

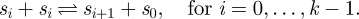

with the initial set containing *k* copies of *s*_0_ . Consider the *x*-copy chemical reaction system **E**_2_^(*x*)^, where *x* is arbitrarily large. We will show that the number of steps needed to produce *s*_*k*_ is exponential in *k*.

Initially, we have *n* = *xk* copies of *s*_0_. Let *n*_*i*_ be the number of copies of *s*_*i*_. Hence, initially, *n*_0_ = *n* and *n*_1_ = · · · = *n*_*k*_ = 0. Note that the chemical reaction system preserves the number of signal molecules present, i.e. at any time, the total number of signal molecules is *n*. Consider the function *F* of *n*_*i*_'s defined as follows:

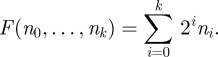



Note that

— initially, the value of *F* is *n*;
— each forward reaction increases the value of *F* by 1 (and each backward reaction decreases it by 1); and
— if a state containing at least one copy of *s*_*k*_ is reached then the value of *F* is at least *n* − 1 + 2^*k*^ .

Hence, the number of steps needed to produce *s*_*k*_ is at least 2^*k*^ − 1.

Note that theorem 3.2 is much stronger than our intuitive notion of crosstalk short-circuiting a computation. It states that with only a linear number of copies, any signal molecule can be produced in at most a quadratic length computation.

We can formalize the intuitive notion of short-circuiting. A system **C** is *x*-*copy-tolerant* if, for all *s* ∈ *S*, the length of the shortest trace to produce *s* in **C** and in **C**^(*x*)^ is the same. A system is *copy-tolerant* if it is *x*-*copy-tolerant* for all *x*.

With that definition, we have the following corollary based on the fact that if a 1-proper chemical reaction system is (*B*_**C**_ + 1)-copy-tolerant, then *s*_end_ can be computed in **C** in the same number of steps as in **C**^(*B*_**C**_^^+1)^, which is polynomial in *B*_**C**_ by theorem 3.2.

**Corollary 3.5.** *For any 1-proper chemical reaction system* **C** = 〈*S*, *ℛ*, *S*_0_, *s*_end_〉 *that is* (*B*_**C**_ + 1)-*copy-tolerant, if there is a computation that produces a given signal molecule* *s*_end_ *in* **C** *then there is a computation that produces* *s*_end_ *in* **C** *in* *O*((*B*_**C**_)^2^) *steps*.

Informally, this implies that any chemical reaction system that is robust in a multi-copy setting cannot have signal molecules whose production requires a computation length exponential in the size of the system.

## 4. Conclusions

In this paper, we have introduced the concept of recycling, or molecule reuse, in strand displacement systems and chemical reaction systems. Our *n*-bit GRAY counters effectively use recycling to step through 2^*n*^ states while consuming, or wasting, molecules whose total number of bases is *O*(*n*^3^). Our GRAY counter strand displacement constructions also introduce the use of a *mutex* strand to ensure that higher level chemical reactions are executed atomically. Finally, we show limits to recycling: for example, signal molecules representing the final state of our *n*-bit counter can be generated using just *O*(*n*^2^) reactions when **Θ**(*n*) copies of the initial signal molecules share the same volume.

One weakness of our counter construction is that the number of distinct domains needed is polynomial in *n*, the number of bits of the counter. In contrast, a QSW binary counter that is implemented via the stack machine of Qian *et al.* [11] uses just a constant number of domains independent of *n*. Is it possible to construct an *n*-bit counter that combines the best of the GRAY and QSW counters, i.e. generates waste that is polynomial in *n* and uses *O*(1) distinct domains? More generally, can *all* computation be realized by strand displacement systems whose waste and haste are within a (small) polynomial factor of the space and time of the computation? Our negative result raises the question as to whether there are alternative strand displacement realizations of certain chemical reaction system classes that generate little waste, say logarithmic in the computation length, and that also behave correctly in the multi-copy setting. We will investigate these questions in future work.

## Footnotes

1 ‘Atomic’ is standard computer science terminology for something that occurs as if all at once, hearkening back to the original Greek etymology of an atom as an indivisible unit. Reasoning about chemical reactions as computational processes can unfortunately result in clashes in terminology.

2 A computation may have multiple final states. To model this situation, we can let *s*_end_ be produced in all final reactions, in addition to any other signal molecules that may indicate the result of the computation.
